# Supplementary material for: Splice-Junction-Based Mapping of Alternative Isoforms in the Human Proteome
Source: Cell Rep. Author manuscript; Available in PMC 2020 Jan 15. (PMC6961840; doi:10.1016/j.celrep.2019.11.026)
Supplement: 2 [file NIHMS1546469-supplement-2.zip › DF1/PXD009737/TestisLysC/TestisLysC_4_SUN1_GEPVAFETSASFSPPQCVPENTGYTYALSSSYSSDALDFETEHK.pdf]

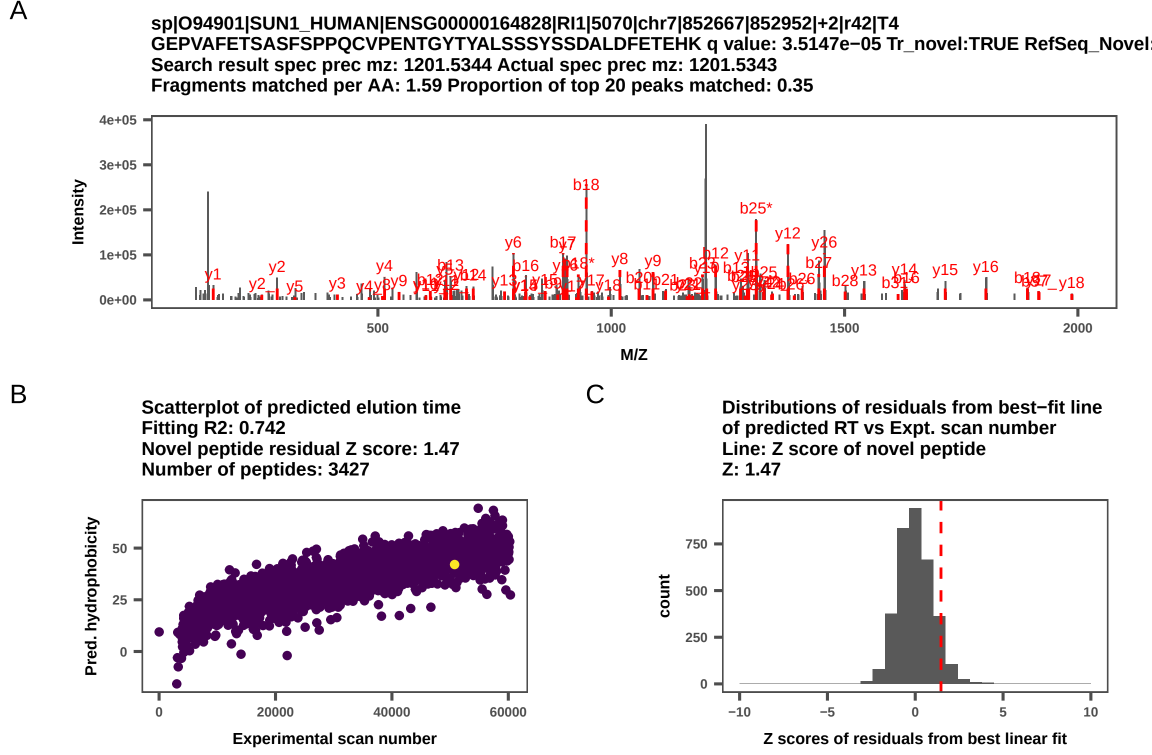
A
sp|O94901|SUN1_HUMAN|ENSG00000164828|RI1|5070|chr7|852667|852952|+2|r42|T4
GEPVAFETSASFSPPQCVPENTGYTYALSSSYSSDALDFETEHK q value: 3.5147e−05 Tr_novel:TRUE RefSeq_Novel:
Search result spec prec mz: 1201.5344 Actual spec prec mz: 1201.5343
Fragments matched per AA: 1.59 Proportion of top 20 peaks matched: 0.35
Intensity
4e+05
3e+05
2e+05
1e+05
0e+00
M/Z
500
1000
1500
2000
b18
b25*
y12
y26
y6
b17
y7
y8
y9
b12
y11
b27
y13
y14
y15
y16
y1
y2
y4
y3
y5
y9
b16
b13
y18
b28
B
Scatterplot of predicted elution time
Fitting R2: 0.742
Novel peptide residual Z score: 1.47
Number of peptides: 3427
Pred. hydrophobicity
50
25
0
0
20000
40000
60000
Experimental scan number
C
Distributions of residuals from best−fit line
of predicted RT vs Expt. scan number
Line: Z score of novel peptide
Z: 1.47
count
750
500
250
0
−10
−5
0
5
10
Z scores of residuals from best linear fit
